# Progressive Modular Rebalancing System and Visual Cueing for Gait Rehabilitation in Parkinson's Disease: A Pilot, Randomized, Controlled Trial With Crossover

**DOI:** 10.3389/fneur.2019.00902

**Published:** 2019-08-29

**Authors:** Mariano Serrao, Francesco Pierelli, Elisabetta Sinibaldi, Giorgia Chini, Stefano Filippo Castiglia, Marina Priori, Dario Gimma, Giovanni Sellitto, Alberto Ranavolo, Carmela Conte, Michelangelo Bartolo, Giuseppe Monari

**Affiliations:** ^1^Department of Medical and Surgical Sciences and Biotechnologies, Sapienza University of Rome, Rome, Italy; ^2^Movement Analysis Laboratory, Policlinico Italia, Rome, Italy; ^3^IRCCS Neuromed, Pozzilli, Italy; ^4^Department of Occupational and Environmental Medicine, Epidemiology and Hygiene, INAIL, Rome, Italy; ^5^IRCCS Don Carlo Gnocchi Foundation, Milan, Italy; ^6^Neurorehabilitation Unit, Department of Rehabilitation, HABILITA Zingonia, Bergamo, Italy

**Keywords:** neurorehabilitation, Parkinson's disease, gait analysis, progressive modular rebalancing system, sensory cues

## Abstract

**Introduction:** The progressive modular rebalancing (PMR) system is a comprehensive rehabilitation approach derived from proprioceptive neuromuscular facilitation principles. PMR training encourages focus on trunk and proximal muscle function through direct perception, strength, and stretching exercises and emphasizes bi-articular muscle function in the improvement of gait performance. Sensory cueing, such as visual cues (VC), is one of the more established techniques for gait rehabilitation in PD. In this study, we propose PMR combined with VC for improving gait performance, balance, and trunk control during gait in patients with PD. Our assumption herein was that the effect of VC may add to improved motor performance induced by the PMR treatment. The primary aim of this study was to evaluate whether the PMR system plus VC was a more effective treatment option than standard physiotherapy in improving gait function in patients with PD. The secondary aim of the study was to evaluate the effect of this treatment on motor function severity.

**Design:** Two-center, randomized, controlled, observer-blind, crossover study with a 4-month washout period.

**Participants:** Forty individuals with idiopathic PD in Hoehn and Yahr stages 1–4.

**Intervention:** Eight-week rehabilitation programs consisting of PMR plus VC (treatment A) and conventional physiotherapy (treatment B).

**Primary outcome measures:** Spatiotemporal gait parameters, joint kinematics, and trunk kinematics.

**Secondary outcome measures:** UPDRS-III scale scores.

**Results:** The rehabilitation program was well-tolerated by individuals with PD and most participants showed improvements in gait variables and UPDRS-III scores with both treatments. However, patients who received PMR with VC showed better results in gait function with regard to gait performance (increased step length, gait speed, and joint kinematics), gait balance (increased step width and double support duration), and trunk control (increased trunk motion) than those receiving conventional physiotherapy. While crossover results revealed some differences in primary outcomes, only 37.5% of patients crossed over between the groups. As a result, our findings should be interpreted cautiously.

**Conclusions:** The PMR plus VC program could be used to improve gait function and severity motor of motor deficit in individuals with PD.

**Clinical Trial Registration**: www.ClinicalTrials.gov, identifier NCT03346265.

## Introduction

Gait disturbances are considered one of the most disabling aspects of Parkinson's disease (PD) (1, 2) and can strongly impact a patient's independence and quality of life (3). The mechanisms underlying gait deficits are multi-factorial and often caused by a multisystem lesion involving both dopaminergic and non-dopaminergic mechanisms (4–9), related not only to bradykinesia, rigidity, abnormal trunk control, and postural instability (1, 2, 10–12) but also to cognition (13–16).

Perhaps even more significant than clinical and functional impacts of gait impairment, this pathological consequence of PD also determines social and economic costs due to falls and trauma. The significance of treating gait disturbances is reinforced by prior work showing that gait outcomes are related to longevity (17), cognitive decline (18), and adverse events (19). Therefore, rehabilitative interventions for the treatment or attenuation of gait impairments should be one of the primary foci of inpatients with PD.

One of the longest studied and most documented techniques for gait rehabilitation in PD is the use of sensory cueing (20, 21). Several studies have shown improvement in electromyographic and spatiotemporal parameters of gait in PD patients undergoing gait training with auditory, visual, and tactile cues (22–27).

While the mechanisms responsible for the improvements in gait due to sensory cueing are not fully understood, it is believed that individuals with PD have lower activity in certain brain areas that are responsible for the internal pathways needed to implement automatic and sequential movements (28). For instance, during sensory cueing for walking with visual cues (VC), patients with PD likely focus their attention on the discrete goal of each foot, hit a VC placed on the floor, and then use exteroceptive information (i.e., position of the next foot placement location) to plan each step individually at a cortical level (25). In addition to gait-oriented training (21, 29), several different approaches using exercise therapy have been proposed aiming at improving mobility, muscular strength, resistance, balance, and aerobic conditioning, and endurance and axial alignment (30–33). This heterogeneous mix of rehabilitation approaches also revealed indirect improvements in gait function (32, 33). Furthermore, some cognitive rehabilitative techniques including action observation therapy and motor imagery have recently been proposed to facilitate gait and motor performance in patients with PD (34).

These previous findings then suggest that a variety of rehabilitative procedures can be effective and the optimal type of physiotherapy activity has not yet been determined (32, 33). As such, a single multifaceted, structured, and comprehensive rehabilitative approach, acting on the different aspects of motor control (e.g., balance, muscle strength, flexibility, trunk and joint mobility, and muscle endurance) is needed for treating gait disturbances and motor impairment in patients with PD. The European Physiotherapy Guidelines for PD has proposed specific areas of intervention to address this need. These guidelines propose a series of exercises that can be combined into one rehabilitation program; however, they are currently not functionally connected to each other in a single structured rehabilitation procedure.

The progressive modular rebalancing (PMR) system is an exercise-based therapy based on proprioceptive neuromuscular facilitation (PNF) principles (35–37). PNF was further developed into an alternate approach (38) mainly focusing on trunk mobility, strength, endurance, and functional connection with proximal muscles. It may be particularly appropriate for patients with PD in whom the abnormal activation of trunk rotator and extensor muscles, trunk motion, ability to roll over on the bed, and axial rigidity are all associated with a high risk of falls (39, 40). PMR proposes a trunk-specific exercise program that is preliminary and preparatory for gait exercises. The link between trunk and gait exercises is proposed as proximal movement stimulation, which is performed through rhythmic stimulation of scapular and pelvic girdle movements. PMR gait exercises are performed with a focus on interlimb coordination and reactive postural control and are preceded by exercises aimed at strengthening the bi-articular musculature involved in walking through PNF patterns (41).

In this regard, the European Physiotherapy Guidelines for PD reported only one trial on trunk muscle strength training for gait improvement and recommended the identification and correction of trunk muscle weakness in the design of rehabilitation programs, as traditional abdominal crunches alone were not effective (42).

In the present study, a rehabilitation program was proposed for patients with PD based on the combination of PMR and VC aimed at improving gait performance by improving balance and trunk control during gait movement. Our hypothesis was that the effect of VCs may interact with improved motor performance induced by the PMR treatment. Specifically, it is hypothesized that patients may improve their bi-articular hip muscle function and trunk and balance control through the PMR system and thus better exploiting the information (spatial and temporal) delivered by the VC, resulting in improvements in specific gait parameters, joint kinematics, and trunk motion.

The primary aim of this pilot trial was to establish whether an 8-week PMR exercise program focused on improving gait function in addition to VC training in people with PD was more effective than a same-duration program of conventional physiotherapy including VC as recommended by European Physiotherapy Guidelines for PD.

The secondary aim was to evaluate the effect of these interventions on the disease severity.

## Materials and Methods

### Participants

Sixty individuals with idiopathic PD admitted for outpatient rehabilitation were assessed for eligibility at two rehabilitation centers between May 2015 and December 2017. Forty subjects were ultimately included in the study. The inclusion criteria were as follows: (i) diagnosis of idiopathic PD according to UK bank criteria (43), (ii) Hoehn and Yahr stages 1–4 (44), and (iii) UPDRS-III gait sub-score of 1 or higher (45). All patients were in a stable drug program and acclimated to their current medication use for at least 2 weeks. Exclusion criteria were as follows: (i) cognitive deficits (defined as scores of <26 on the Mini-Mental State Examination), (ii) moderate or severe depression (defined as scores of >17 on the Beck Depression Inventory), and (iii) orthopedic and/or other gait-influencing diseases such as other neurological diseases, arthrosis, or total hip joint replacement.

A mandatory requirement for inclusion in the study was also the ability to walk independently for at least 8 m along the laboratory pathway without showing freezing of gait.

**Figure 1:**
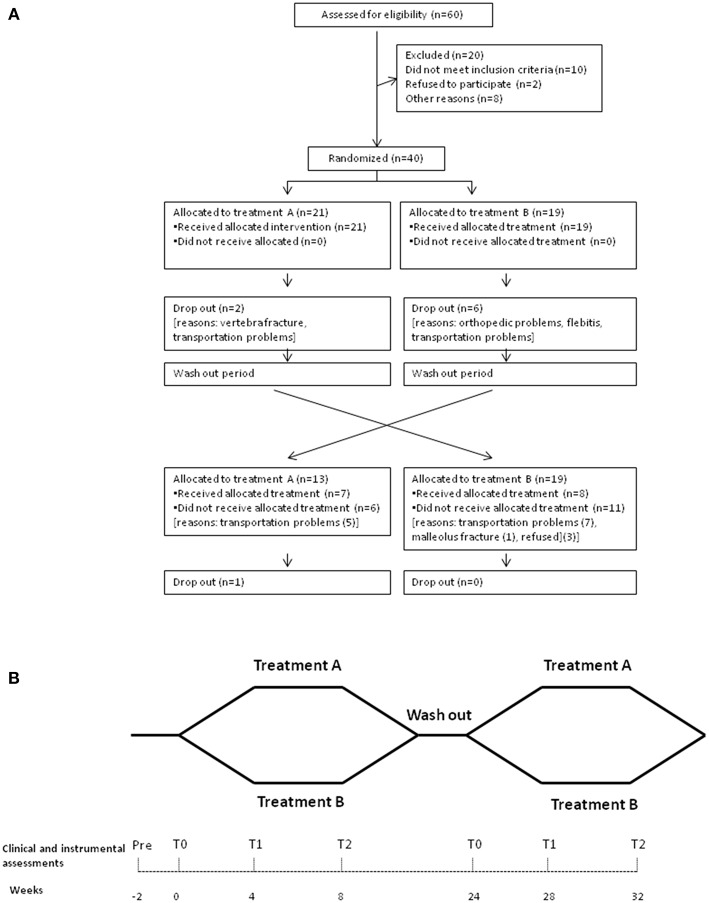
Outline of the study design. **(A)** The flow diagram of the patients enrolled for the study. **(B)** When both the clinical and the instrumental assessments were performed: at baseline, before rehabilitative treatment (T0), at 4 weeks after the beginning of the rehabilitative treatments (T1), and at 8 weeks (at the end of rehabilitation program) (T2).

The study was approved by the ethics committee of Hospital Policlinico Umberto I of Rome/Sapienza University of Rome (Approval Number: 2346454) and patients provided written informed consent. All procedures conformed to the Helsinki Declaration. The study was registered with ClinicalTrials.gov (clinical trial identifier: NCT03346265). The detailed participant flow is shown in Figure 1A.

### Study Design

This was a pilot, two-center, randomized, blind observer, controlled trial with crossover, following the recommendations of the Consolidated Standards of Reporting Trials (46).

Subjects participated in a baseline assessment session (T0, before rehabilitative treatment) and were randomly allocated to an 8-week rehabilitation program (A or B) followed by a 4-month washout period (patients did not have to perform any rehabilitative treatment), after which patients who received treatment A switched to treatment B and vice versa. A computer-based randomization schedule was used. All patients were assessed at the same center. Randomization was stratified according to a block of 20 numbers, so that each block comprised 10 patients randomly assigned to treatment A and 10 patients assigned to treatment B. Since all the subjects were evaluated at the same center, allocation was performed by an investigator not involved in subject recruitment or assessment at the end of the baseline assessment.

Both clinical (neurological visit and scale administration) and instrumental (gait analysis) assessments were performed at baseline, before rehabilitative treatment (T0), 4 weeks after the beginning of the rehabilitative treatments (T1), and at 8 weeks (at the end of rehabilitation program) (T2) (Figure 1B). Medication use remained constant throughout the study period, and all the treatments were performed at the same time of the day for each patient during the ON phase.

Participants were asked to maintain their daily pre-enrollment activity level.

Assessors, for both clinical and instrumental evaluations, were blinded to the allocation of treatment.

### Intervention

The exercise program was conducted three times per week for 60 min over an 8-week period. Physical therapists with expertise in PD administered the exercise programs (ES, SFC, MP, DG, and GS).

#### Treatment A

Treatment A was performed 3 days/week. Each session was 60 min in duration and consisted of a combined exercise program of 40 min of PMR (47) and 20 min of gait training performed with VC.

Each session was divided into muscular stretching exercises, aiming at increasing the step length and the mobility of the trunk, and tailored progressive exercise therapy. Stretching exercises were performed based on the contract–hold–relax principles, and trunk muscles were lengthened. Perception exercises reciprocally activating anterior elevation and posterior depression of both the shoulder and pelvis complex were performed. Trunk strength exercises were performed based on postural steps, moving from the supine to the upright position, and specific extensor muscle recruitment exercises. Recruitment exercises aiming to reach and maintain specific symmetrical positions (like supine bridging or the reverse tabletop pose) were performed by patients presenting camptocormia, and asymmetrical positions (like side sitting or side bridging) were performed by patients presenting with the Pisa syndrome. Physical therapists guided the patients during the walking training by stimulating upper limb movements at the same time. Walk training was also performed for the knees to better recondition reciprocal hip movements.

A further detailed description of the PMR technique with motor patterns is reported in Table 1.

**Table 1:** A detailed description of the progressive modular rebalancing technique with motor patterns.

**STRETCHING EXERCISES**	
** 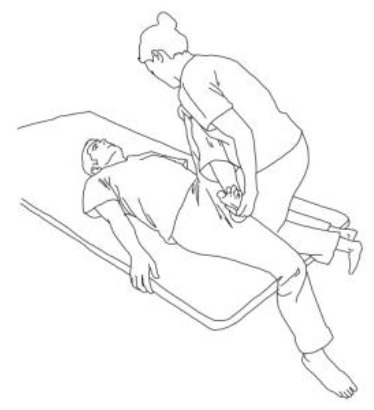 **	** 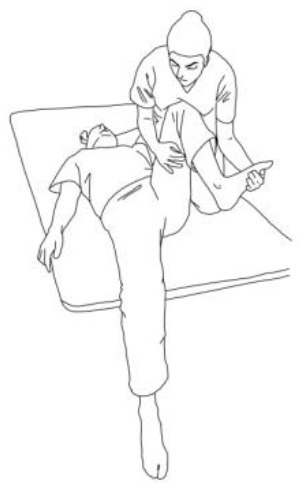 **
**Gluteus medius stretching:** The therapist flexes, adduces, and externally rotates the limb to be stretched. The contralateral leg is extended, adducted, and externally rotated and the knee flexed. The therapist asks to extend, abduct, and internally rotate the hip to be stretched against his body and, after relaxing, gains range of motion 3–5 times.	**Gluteus maximus and adductor magnus stretching:** The therapist flexes, abducts, and internally rotates the hip to be stretched. The contralateral hip is extended, abducted, and internally rotated, the knee flexed. The therapist asks to extend, adduce, and externally rotate the hip to be stretched against his resistance, to hold, and, after relaxing, gains range of motion. Repetitions: 3–5 times.
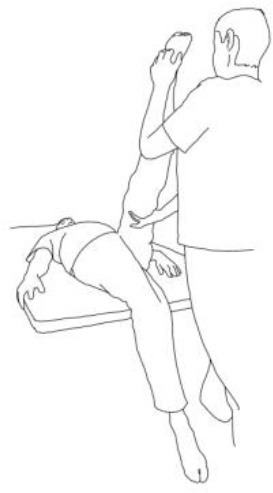	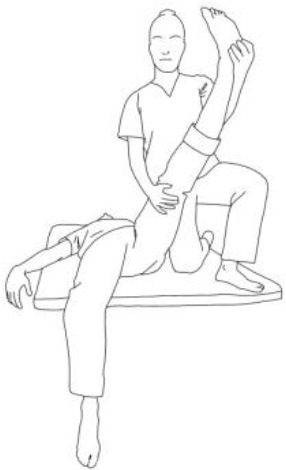
**Biceps femoris stretching:** The therapist flexes, adduces and externally rotates the leg to be stretched with an extended knee. The contralateral leg is extended, adducted, and externally rotated and the knee flexed. The therapist asks to extend, abduct, and internally rotate the hip to be stretched against his body and, after relaxing, gains range of motion 3–5 times.	**Semitendinous and semimembranosus stretching:** The therapist flexes, abducts, and internally rotates the leg to be stretched with an extended knee. The contralateral hip is extended, abducted, and internally rotated, and the knee flexed. The therapist asks to extend, adduce, and externally rotate the hip to be stretched against his resistance, to hold, and, after relaxing, gains range of motion. Repetitions: 3–5 times.
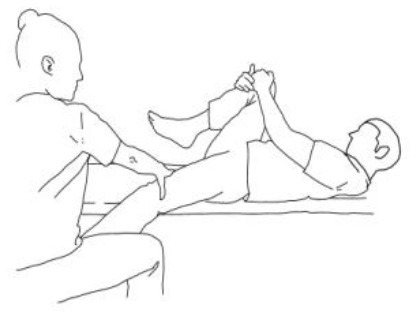	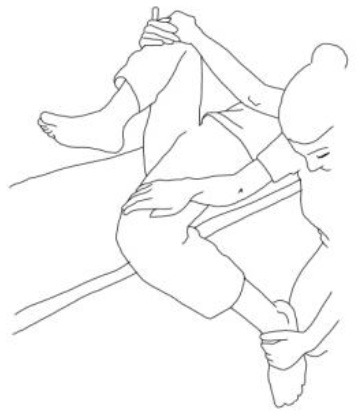
**Iliopsoas stretching:** The therapist extends, abducts, and internally rotates the hip to be stretched, and the knee is extended. The contralateral leg is flexed, abducted, and internally rotated, asking the patient to hold it. The therapists ask to flex, adduce, and externally rotate the hip to be stretched against his resistance, to hold, and, after relaxing, gains range of motion. Repetitions: 3–5 times.	**Quadriceps femoris stretching:** The therapist extends, abducts, and internally rotates the hip to be stretched and flexes the knee. The contralateral leg is flexed, abducted, and internally rotated, asking the patient to hold it. The therapists ask to flex, adduce, and externally rotate the hip and to extend the knee to be stretched against his resistance, to hold, and, after relaxing, gains range of motion by flexing the knee. Repetitions: 3–5 times.
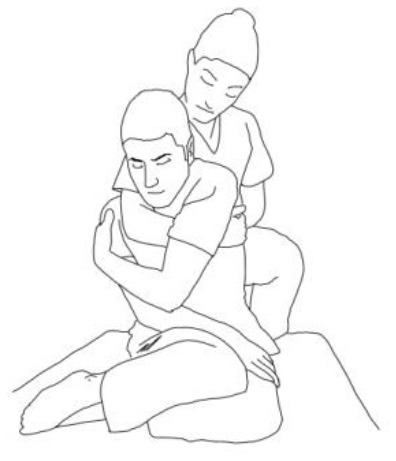	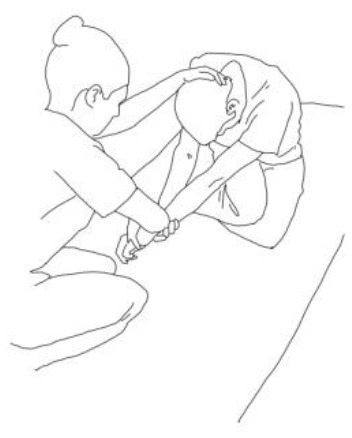
**Rotary torso muscles stretch:** The patient is side sitting, the therapist is behind him and rotates his torso, asks to rotate against him, to hold, and then, after relaxing, gains range of motion toward the concave side. Repetitions: 3–5 times.	**Torso extensor muscles stretch:** The patient is side sitting, the therapist is in front of him and flexes his torso by flexing his head and extending his arms, asks to lift up patient's arms and look at that against his resistance, to hold, and then, after relaxing, gains range of motion toward the concave side. Repetitions: 3–5 times.
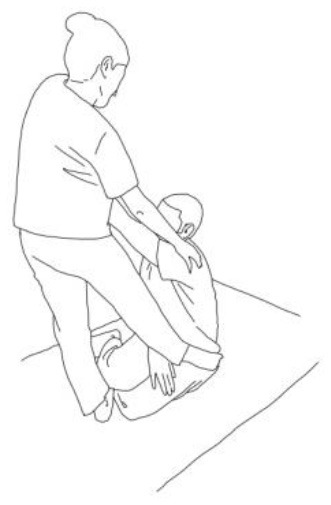	
**Obliquus muscles stretch:** The patient is side sitting. The therapist is standing in front of him and pulls the patient's arm up high and tilts the torso toward the concave side. The therapist asks to extend, abduct, and internally rotate the patient's arm, then to hold, and then, after relaxing, inclines the trunk further toward the concave side. Repetitions: 3–5 times.	
**TRUNK POSTURAL ALIGNMENT EXERCISES**	
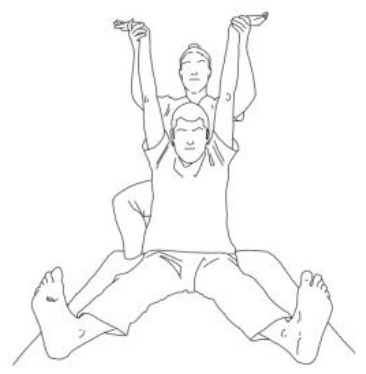	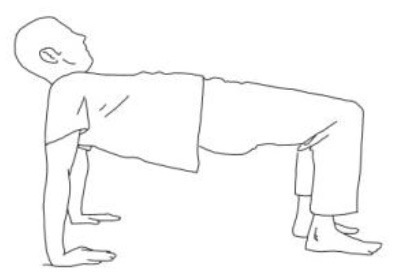
**Exercises for the erector spinae muscles:** The patient is long sitting, the therapist is behind him and asks him to hold an isometric contraction against his resistance at the end of a bilateral flexion–abduction–external rotation pattern for 5 s at least.	**Reverse tabletop pose exercise:** After stretching the shoulder, arm, and lower limb muscles and performing the supine bridge exercise, the goal is to reach and maintain this position with extended and 90° flexed knees, flexed head, and well-adduced shoulder blades.
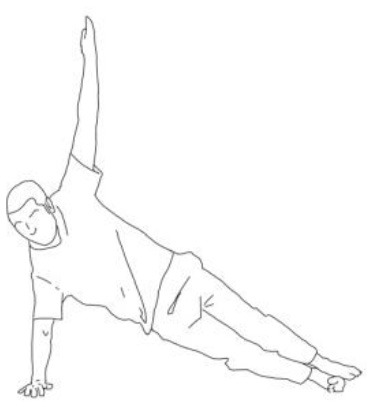	
**Side bridge exercise:** After having stretched out the obliquus muscles of the tilted side and having recruited those of the weakest side, performing this exercise on the weakest side is the goal for patients presenting Pisa Syndrome. The exercise can be performed bearing on the elbow or by flexing the knee too.	
**WALKING TRAINING**	
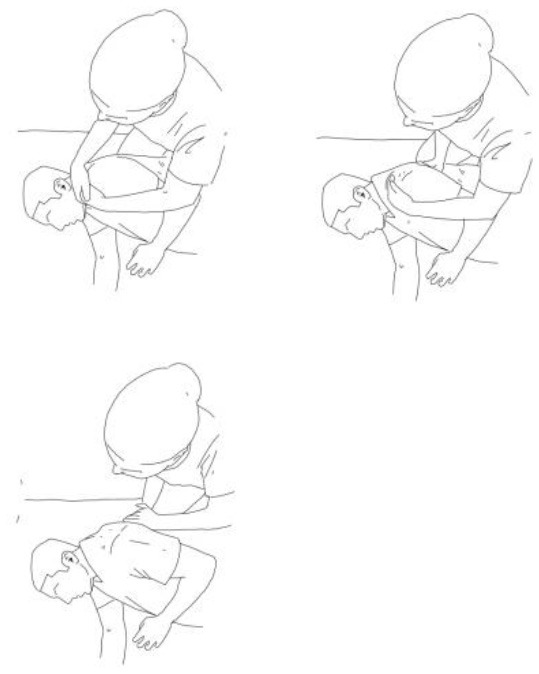	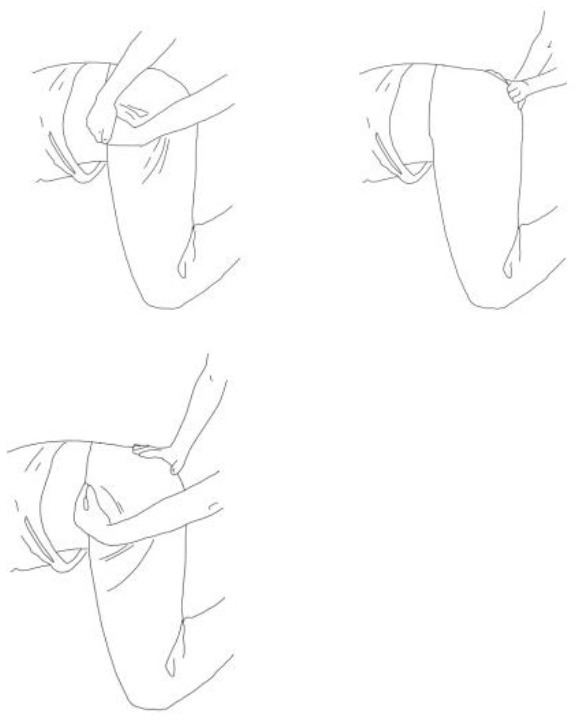
**Stimulation of the movements of the shoulder complex:** The therapists rhythmically ask the patient to anteriorly elevate his shoulder toward his nose or posteriorly depress it by adducing his shoulder blade toward the column. First, the patient has to perceive the passive movement performed by the therapist and then has to perform it actively against resistance. When the patient can perform the two movements, the therapist asks him to reciprocally activate anterior elevation and posterior depression.	**Stimulation of the movements of the pelvic complex:** The therapists rhythmically ask the patient to anteriorly elevate his pelvis toward or posteriorly depress it. First, the patient has to perceive the passive movement performed by the therapist and then has to perform it actively against resistance. When the patient can perform the two movements separately, the therapist asks him to reciprocally activate anterior elevation and posterior depression.
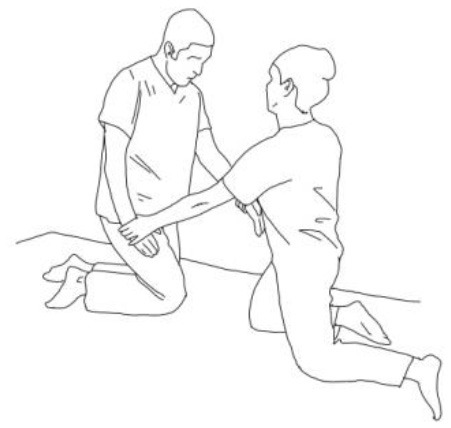	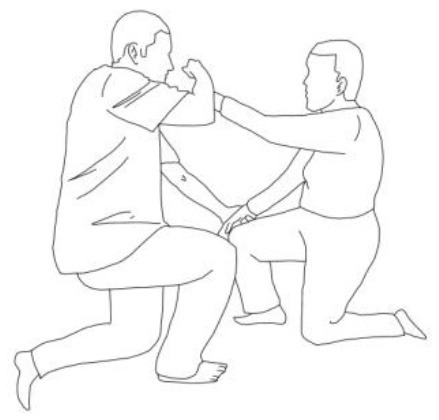
**Walking training on the knees:** The therapists guide the patients in walking on the knees by alternatively stimulating a flexion–adduction–extra rotation pattern of the upper limb on the bearing side and an extension–abduction internal rotation pattern of the upper limb on the swinging side.	**Half-kneeling pose:** The patient has to reach the half-kneeling position from the kneeling one. The therapist facilitates the passage using two upper limb patterns: flexion–adduction–external rotation of the upper limb on the bearing side and extension–abduction internal rotation pattern of the upper limb on the swinging side.
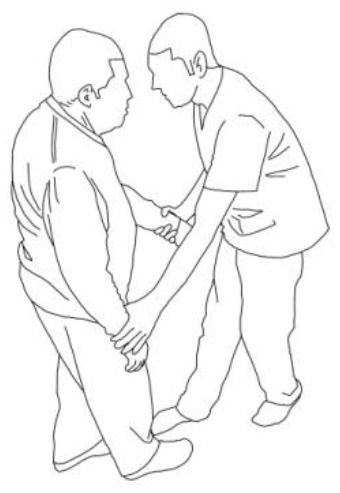	
**Distal guided path:** The therapist facilitates the patient in walking using two upper limb patterns: flexion–adduction–external rotation of the upper limb on the bearing side and extension–abduction internal rotation pattern of the upper limb on the swinging side.	

*Examples of progressive modular rebalancing exercises*.

For the VC training, white parallel transverse lines (white, 800 × 19 mm) were placed on the floor perpendicular to a dark walkway path at intervals equal to 40% of the patient's height. Lines were moved further apart by 0.05 m per stride every 3 or 4 days and did not bend through the chicane. Participants were asked to walk across the lines matching their step length to the interval between the stripes, turn, and return to the start line.

#### Treatment B

Conventional physiotherapy was administered according to the European Physiotherapy guidelines for PD (http://www.appde.eu/european-physiotherapy-guidelines.asp) and focused on the following areas based on the stage of the disease: self-management support, prevention of inactivity and fear of falls, maintaining or improving global motor activities, improvement of physical performance, and improvement in the ability to perform transfer, balance, gait, and manual activities, reduce pain, and delay the onset of physical limitations.

Exercises included the following: standing up from and sitting down on the floor; standing and walking on foam with and without perturbation (pushes and pulls) to the trunk; sitting down onto and rising from a chair (while dual tasking); getting into and out of bed; rolling over in bed; walking and taking large steps with large amplitude arm swings; walking around and over obstacles; walking with sudden stops and changes in walking direction (including walking backwards); walking and maintaining balance while conducting dual tasks (such as talking, carrying an object, or turning head left to right to wall-mounted dots or photos and reporting on what is seen); turning around in open, narrow, and small spaces; and climbing steps or stairs.

The VC training was performed as an integral part of the conventional physiotherapy and consisted of visual white lines placed on the ground in the same way as in treatment A. This was performed three times a week for 30 min as recommended in the European Physiotherapy guidelines. The VCs were discretionally executed by the physiotherapists during the course of each treatment session.

The rehabilitation program is composed of a 60 min session once a day, performed 3 days/week.

Participants within this program were encouraged to progress, based on stated progression criteria. Progression in range of motion exercises, stretching exercises, upper and lower limb strengthening exercises, and improving balance, standing, sitting, transferring, and walking was encouraged in all participants.

#### Gait Analysis

Gait analysis was performed using an eight-ray infrared optoelectronic SMART-DX 500 motion analysis camera and system (BTS, Milan, Italy) with a sampling rate of 300 Hz. The system detected the motion of 22 passive spherical markers, placed over prominent bony landmarks according to the international recommendations and validated biomechanical models (48). Anthropometric data were collected from each participant (48).

Patients were asked to walk barefoot at a comfortable speed along a walkway. As we were interested in natural locomotion, only general qualitative instructions (e.g., “walk at natural speed you would use in your daily life,” “look forward,” and “do not turn or stop”) were provided. The same instructions were given to all participants. Before the recording session, the subjects practiced for a few minutes to familiarize themselves with the procedure. Five trials were recorded for each locomotor task. All patients were recorded in ON state.

We focused on evaluating three important aspects of gait function: gait performance (e.g., speed, step length, hip joint range-of-motion [RoM]), gait balance (gait related-parameters, e.g., step width and double support duration), and trunk control (trunk kinematics).

### Outcome Assessment

#### Primary Outcomes

The following kinematic parameters were measured: stance phase duration (%), double support phase duration (%), cadence (step/min), step length, step width (m), mean speed (m/s), spatial asymmetry index, temporal asymmetry index, hip, knee and ankle flexion–extension RoM and trunk flexion–extension, lateral bending, and rotation RoM.

#### Secondary Outcomes

Disease severity was evaluated using the UPDRS-III (45). A neurologist with expertise in movement disorders and blinded to patients' allocation administered the UPDRS scale.

### Statistical Analysis

A power analysis using the G^*^Power computer program (49), based on preliminary data from the T1 assessment (50) indicated a total sample of 24 participants to detect medium effects (*d* = 0.5) with 80% power using an unpaired *t*-test between means of α = 0.05. Due to the number of gait parameters considered as the primary outcomes of this pilot study, we calculated the sample size according to Whitehead et al. (51), who identified a conservative minimum sample size of 20–40 subjects for a pilot trial. Thus, we chose to consider a sample size ranging from a minimum of 24–40 subjects.

Intention-to-treat analysis (ITT) was conducted, with the ITT population defined as all randomized patients who provided at least one baseline efficacy assessment and attended at least one treatment session.

The Shapiro-Wilk and Levene tests were used to assess normality and homogeneity of variance, respectively, for all measures. Baseline characteristics were compared between the groups using either a Student *t*-test (parametric data) or Mann–Whitney *U*-test (non-parametric data) or, for categorical variables, using the Fisher exact test.

We assessed the effect of the rehabilitative treatments on both the primary and secondary outcomes through an ANOVA mixed-effect model taking into account longitudinal repeated measures including the effect of time (T0–T2) within each treatment group and interaction between time and intervention. Missing values were imputed with the last observation carried forward (i.e., baseline, intermediate evaluation). Greenhouse-Geisser correction was applied, when deemed necessary, to circumvent violations of sphericity (i.e., inequalities in the variance of the differences between factors). The Bonferroni correction for multiple testing was applied for pairwise comparisons to account for the familywise error rate.

A crossover design was used to reduce both the impact of inter-individual variability by exposing each subject to two different interventions and the effect of the disease progression by exposing subgroups to different treatment sequences.

Furthermore, a 4-month rest period (wash-out) between the rehabilitative treatments was introduced to reduce a potential carryover effect and reproduce a hypothetical basal condition after the former intervention. To test for possible carryover effects, we calculated the sum of the values measured in the two periods for each subject and compared across the two sequence groups using a test for independent samples.

Statistical significance was set at *p* < 0.05 for two-sided tests, and all analyses were performed using SPSS 20.0 (IBM SPSS).

## Results

Twenty patients (12%) of the total 60 identified were not enrolled, as they did not meet the inclusion criteria, or declined to participate. Forty patients consented to participate and were enrolled (Figure 1A, Table 2A). According to the H–Y classification, there were 11 patients in stage 1, 13 patients in stage 2, 12 patients in stage 3, and 4 patients in stage 4. Eight of these patients failed to complete T2 and thus were considered as having missing values and inputted with the intermediate observation carried forward (two patients in group A and six patients in group B) (Figure 1A). A total of 32 patients completed the 8-week treatment (treatment adherence: 90.5% in Group A, 68.4% in Group B; *p* > 0.05 for the difference in primary endpoint). The assessments from the eight patients who dropped out were input forward in the final analysis. All patients were taking oral administrations of levodopa (18 patients), dopamine agonists (5 patients), or both (17 patients). No significant differences in demographics were noted between groups at T0 (all, *p* > 0.05) or in clinical characteristics, UPDRS-III, H–Y scale, and total Levodopa Equivalent Dose (LED) (all, *p* > 0.05) (Table 2A).

**Table 2:** **(A)** summarizes complete patient anthropometric and clinical characteristics (mean ± standard deviation).

**Table 2A**	**Anthropometric and clinical characteristics**							
**Group**	**Gender**	**Age (years)**	**Weight (kg)**	**Height (m)**	**Most affected side**	**Disease duration (years)**	**Modified H–Y**	**Total LED**
A	10F/11M	68.857 ± 8.627	69.808 ± 11.559	1.623 ± 0.080	8r/7l/6bil	8.952 ± 4.899	2.9 ± 0.9	593.7 ± 331.5
B	8F/11M	71.158 ± 7.522	75.463 ± 13.735	1.606 ± 0.071	5r/5l/9bil	8.536 ± 3.508	2.9 ± 1.2	623.5 ± 328.6
**Table 2B**	**Gait variables analysis**							
		**Time evaluation**			**Main effect (time)**		Main effect (time^*^treatment)	
**Parameter**	**Treatment**	**T0**	**T1**	**T2**	***F***	***p***	***F***	***p***
Speed (m/s)	A	0.743 ± 0.258	0.918 ± 0.210	0.952 ± 0.199	*F*_(2, 76)_ = 10.664	**<0.001**	*F*_(2, 76)_ = 15.075	**<0.01**
	B	0.736 ± 0.305	0.726 ± 0.337	0.714 ± 0.349				
r stance duration (% cycle)	A	63.962 ± 4.786	61.857 ± 3.008	61.614 ± 2.905	*F*_(1.542, 58.591)_ = 2.070	0.146	*F*_(1.542, 58.591)_ = 7.913	**0.004**
	B	64.721 ± 3.853	65.332 ± 5.016	65.447 ± 5.193				
l stance duration (% cycle)	A	63.809 ± 3.796	61.633 ± 2.854	61.576 ± 2.855	*F*_(1.491, 56.648)_ = 5.315	**0.014**	*F*_(1.491, 56.648)_ = 5.253	**0.014**
	B	64.926 ± 3.984	65.021 ± 5.414	64.826 ± 5.573				
r doub. supp. duration (% cycle)	A	14.148 ± 4.722	11.790 ± 2.917	11.481 ± 2.618	*F*_(1.589, 60.378)_ = 2.071	0.144	*F*_(1.589, 60.378)_ = 7.841	**0.002**
	B	15.047 ± 3.757	15.542 ± 5.287	16.126 ± 5.877				
l doub. supp. duration (% cycle)	A	13.814 ± 3.953	11.781 ± 2.625	11.676 ± 2.885	*F*_(1.710, 64.986)_ = 2.329	0.113	*F*_(1.710, 64.986)_ = 4.492	**0.019**
	B	14.732 ± 4.195	14.847 ± 5.360	15.284 ± 6.401				
Spatial asymmetry	A	0.086 ± 0.092	0.065 ± 0.044	0.054 ± 0.048	*F*_(1.544, 58.669)_ = 3.470	**0.049**	*F*_(1.544, 58.669)_ = 0.551	0.535
	B	0.133 ± 0.098	0.086 ± 0.007	0.102 ± 0.078				
Temporal asymmetry	A	0.026 ± 0.022	0.026 ± 0.025	0.027 ± 0.024	*F*_(2, 76)_ = 0.016	0.853	*F*_(2, 76)_ = 0.103	0.902
	B	0.027 ± 0.018	0.026 ± 0.020	0.030 ± 0.026				
Cadence (step/min)	A	102.988 ± 17.050	110.196 ± 11.729	110.398 ± 13.082	*F*_(1.625, 61.742)_ = 1,843	0.173	*F*_(1.625, 61.742)_ = 5.523	**0.010**
	B	100.11 ± 15.646	98.507 ± 18.335	97.802 ± 18.573				
r step length (m)	A	0.389 ± 0.103	0.449 ± 0.085	0.471 ± 0.071	*F*_(1.654, 62.843)_ = 18.116	**<0.001**	*F*_(1.654, 62.843)_ = 3.598	**0.041**
	B	0.366 ± 0.122	0.385 ± 0.139	0.398 ± 0.144				
l step length (m)	A	0.391 ± 0.099	0.463 ± 0.078	0.485 ± 0.065	*F*_(1.688, 64.152)_ = 15.698	**<0.001**	*F*_(1.688, 64.152)_ = 8.786	**0.001**
	B	0.403 ± 0.122	0.406 ± 0.142	0.419 ± 0.151				
Step width (m)	A	0.165 ± 0.019	0.171 ± 0.022	0.174 ± 0.025	*F*_(1.550, 58.890)_ = 0.094	0.863	*F*_(1.550, 58.890)_ = 4.265	**0.027**
	B	0.165 ± 0.020	0.160 ± 0.020	0.158 ± 0.019				
Trunk flexion–extension RoM (°)	A	4.339 ± 0.546	4.260 ± 0.063	5.172 ± 0.848	*F*_(1.499, 59.961)_ = 20.241	**<0.001**	*F*_(1.499, 59.961)_ = 10.142	**0.001**
	B	4.276 ± 0.155	4.215 ± 0.092	4.392 ± 0.400				
Trunk bending RoM (°)	A	3.844 ± 0.532	4.183 ± 0.043	4.698 ± 0.774	*F*_(1.572, 59.728)_ = 17.530	**<0.001**	*F*_(1.572, 59.728)_ = 6.352	**0.006**
	B	4.096 ± 0.077	4.142 ± 0.053	4.304 ± 0.382				
Trunk rotation RoM (°)	A	7.797 ± 1.612	8.325 ± 0.200	9.486 ± 2.644	*F*_(1.237, 47.012)_ = 5.523	**<0.001**	*F*_(1.237, 47.012)_ = 1.083	0.318
	B	7.578 ± 0.345	8.367 ± 0.194	8.840 ± 0.589				
r hip RoM (°)	A	32.175 ± 1.417	31.668 ± 0.348	33.885 ± 1.954	*F*_(1.424, 54.108)_ = 47.544	**<0.001**	*F*_(1.424, 54.108)_ = 5.909	**0.010**
	B	30.785 ± 0.717	31.994 ± 0.382	33.737 ± 1.465				
l hip RoM (°)	A	32.591 ± 2.773	31.669 ± 0.251	35.567 ± 2.646	*F*_(2, 76)_ = 42.921	**<0.001**	*F*_(2, 76)_ = 6.245	**0.003**
	B	31.066 ± 0.565	32.046 ± 0.386	33.665 ± 1.394				
r knee RoM (°)	A	45.199 ± 2.355	45.272 ± 0.261	45.155 ± 2.292	*F*_(2, 76)_ = 4.090	0.21	*F*_(2, 76)_ = 5.582	**0.023**
	B	44.271 ± 0.682	45.556 ± 0.336	45.593 ± 0.667				
l knee RoM (°)	A	44.648 ± 2.046	45.913 ± 0.355	47.875 ± 2.632	*F*_(2, 76)_ = 54.147	**<0.001**	*F*_(2, 76)_ = 0.298	0.744
	B	44.599 ± 0.817	46.261 ± 0.432	47.796 ± 1.364				
r ankle RoM (°)	A	23.237 ± 1.697	23.039 ± 0.141	23.849 ± 1.587	*F*_(2, 76)_ = 14.857	**<0.001**	*F*_(2, 76)_ = 2.321	0.105
	B	22.572 ± 0.318	23.054 ± 0.208	24.001 ± 0.883				
l ankle RoM (°)	A	22.967 ± 1.969	23.128 ± 0.082	24.333 ± 1.516	*F*_(2, 76)_ = 25.302	**<0.001**	*F*_(2, 76)_ = 0.682	0.509
	B	22.329 ± 0.348	22.998 ± 0.110	24.071 ± 0.845				
UPDRS III	A	14.619 ± 5.334	13.810 ± 5.419	13.429 ± 5.287	*F*_(1.622, 61.629)_ = 6.556	**0.005**	*F*_(1.622, 61.629)_ = 3.258	0.606
	B	16.882 ± 7.859	15.118 ± 6.879	13.524 ± 5.259				

This table **(B)** shows comparisons (mixed ANOVA) between the baseline (T0), T1, and T2 follow-up evaluations and between the two groups of patients (A and B). Main effects of time and time^*^treatment are reported. Bold p-values denote statistically significant differences.

### Primary Outcomes (Gait Parameters)

A significant main effect of time^*^group interaction was found in speed, right and left stance duration, right and left double support duration, left step length, cadence, step width, spatial asymmetry, right and left hip RoM, right and left knee RoM, right and left ankle RoM, trunk flexion–extension, and trunk bending (Table 2B).

**Figure 2:**
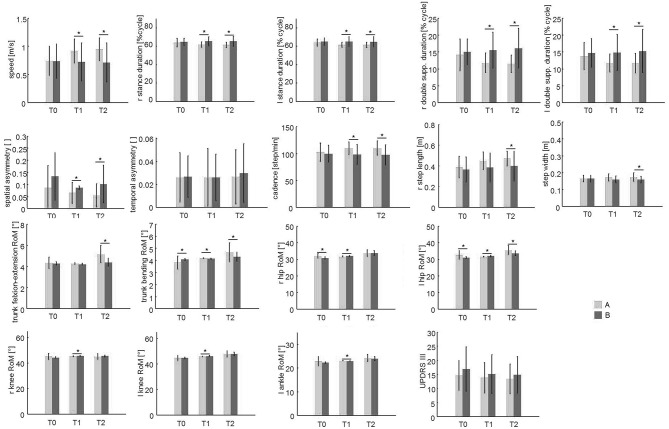
The spatio-temporal parameters and trunk and lower limb joint kinematics at the baseline (T0), T1, and T2 evaluations. This figure shows the mean and the standard deviation values of the 21 patients of group A (which performed PMR + sensory treatment) compared to the 19 patients of group B (which performed standard physiotherapy treatment) at the three evaluations (T0, T1, T2). Asterisks (*) denote statistically significant differences.

*Post hoc* analysis revealed no significant differences between groups at T0 for almost all variables with the exception of right and left hip RoM. Significant improvements in almost all gait parameters were found in Group A compared to Group B at both or either T1 or T2, except for right ankle RoM and trunk rotation, which were not different between the two treatment groups (Figure 2).

A significant main effect of time was found in speed, left stance duration, spatial asymmetry, trunk flexion–extension, trunk bending, trunk rotation, right and left step length, right and left hip RoM, right and left knee RoM, and right and left ankle RoM (Table 2B).

*Post hoc* analysis revealed significant improvements at both or either T1 and T2 compared to T0 (Figure S1) in speed, left stance duration, right and left step length, trunk flexion–extension RoM, trunk bending, trunk rotation, right and left hip RoM, right and left knee RoM, and right and left ankle RoM.

### Secondary Outcomes (UPDRS Scores)

No significant time^*^group interaction was found on UPDRS-III (Figure 2, Table 2B).

A significant main effect of time was found on the UPDRS-III score (Table 2B). *Post-hoc* analysis revealed significant improvement (lower values) of the UPDRS-III scores at T2 compared to that at T0 (Figure S1). UPDRS-III score changed from 15.7 points at T0 to 14.4 at T1 to 14.1 at T2 (Table 2B).

### Patient Crossover

In this study, 15 patients (37.5%) crossed over between the groups (8 patients from A to B and 7 patients from B to A) (Figure 1A).

There was no difference in demographic characteristics between treatment groups (sex: 5F/3M vs. 4F/3M; age: 71.8 ± 7.5 vs. 67.7 ± 6.3; weight: 66.3 ± 7.2 vs. 73.7 ± 17.1; height: 1.60 ± 0.06 vs. 1.60 ± 0.05; disease duration: 7.0 ± 4.4 vs. 9.4 ± 3.4; respectively, all, *p* > 0.05). No significant differences were revealed at the baseline evaluation between treatment A and treatment B groups in the UPDRS-III (12.3 ± 6.2 vs. 12.6 ± 4.6, *p* > 0.05) and H–Y (2.4 ± 0.8 vs. 1.9 ± 1.2, *p* > 0.05) scale scores.

No carryover effect was found for either gait variables or UPDRS scores (*p* > 0.05).

**Figure 3:**
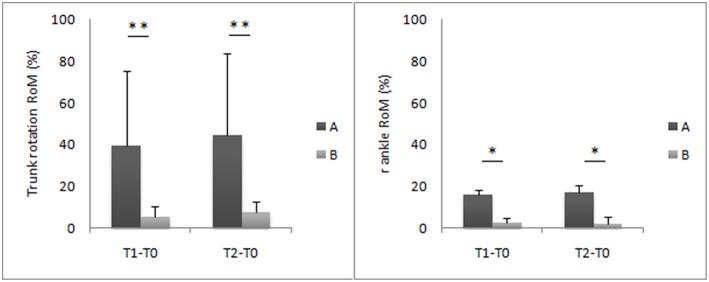
Trunk and ankle joint kinematic improvements at T1 and T2 evaluations. This figure shows the mean percentage difference and the standard deviation values of the eight patients of group A compared to the seven patients of the group B. Asterisks denote statistically significant differences (*p < 0.05, **p < 0.01).

Due to the small number of subjects who crossed over, the non-parametric Mann–Whitney *U*-test was used to compare gait parameters, expressed as a percentage difference from the after-washout baseline values between the two treatments, at T1 and T2. A significantly greater improvement in trunk rotation RoM (T1: Cohen's *d* = 1.28; T2: Cohen's *d* = 1.36) and right ankle RoM (T1: Cohen's *d* = 6.50; T2: Cohen's *d* = 5) was found with treatment A when compared with treatment B (Figure 3). No significant differences were found for the other parameters.

## Discussion

The present findings showed that a rehabilitative approach based on PMR plus VC for rehabilitation of gait function in people with PD appears to be more beneficial when compared to conventional physiotherapy executed according to European guidelines. Specifically, these findings can be summarized as follows: (i) both treatments improved gait function and motor function severity; (ii) patients who received PMR with VC presented with better results in gait performance (increased step length, speed, and joint kinematics), gait balance (increased step width and double support duration), and trunk control (increased trunk motion) than those who received conventional physiotherapy; and (iii) although only 37.5% of patients crossed over between the groups, there still were some differences in the primary outcomes.

**Figure 4:**
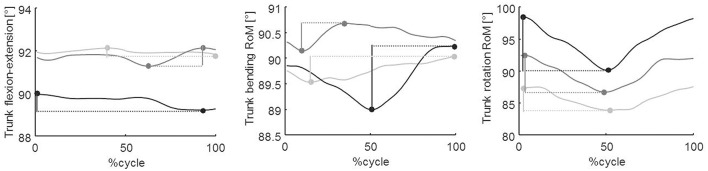
Trunk kinematics in the three spatial planes. From left to the right: sagittal, frontal, and transverse planes, respectively. This figure shows trunk angles at baseline (T0, light gray line) and at T1 (dark gray line) and T2 (black line) follow-up evaluations in a representative patient. Data were normalized to the cycle duration and represented as a percentage of the gait cycle. In the first, second, and third panels, the vertical segments represent the flexion–extension, bending, and rotation RoM, respectively.

The results are in concordance with previous data from Cochrane and systematic reviews, which reported that patients with PD showed a short-term positive effect on gait and balance functions and on motor function severity from several different rehabilitative techniques (32, 33). However, as revealed in this study, PMR plus VC seems to be significantly better than conventional physiotherapy in improving almost all performance-related gait parameters, balance-related gait parameters, and trunk motion (Figures 2–4, Table 2). Thus, the PMR technique should be considered in addressing gait function in patients with PD. The European Physiotherapy Guideline for Parkinson's disease (52) identified five core areas in which a rehabilitation program should lead to improvements, depending on the patient's cognitive condition and the stage of the disease: physical capacity, weight transfer, manual activities, balance, and gait. Improvements in these areas can be expected to lead to improved performance in activities of daily living. However, the interventions used previously are largely heterogeneous (e.g., stretching, muscle strengthening, balance, postural exercises, occupational therapy, cueing, treadmill training) and, taken as a whole, are not part of a unique and directed rehabilitation system. In addition, presently, there is still no consensus about the optimal approach for PD patients (33). PMR is a context-adaptable rehabilitation method in which both patient assessment and exercise are trunk-centered. This aims to progressively recover first the control of the trunk and then its relationship with the limbs, combining them in multiple motor schemes performed in different postural configurations (see Table 1). Notably, in addition to an improvement in the gait spatiotemporal parameters and joint kinematics, we also found a significant improvement in trunk motion (Figure 4). Since a high percentage of patients with PD show postural abnormalities and poor trunk control (8), which predispose them to a high risk of fall (53), special attention should be paid to these aspects of motor control. Indeed, the head and trunk comprise 60% of the overall mass of the body. Thus, one's ability to precisely coordinate trunk movements during walking contributes significantly to creating a more energy-efficient gait pattern, coupling action of the trunk, and pelvis as a resonating pendulum and reducing overall momentum (54). PMR plus VC also showed better improvement in balance-related gait parameters (i.e., step width and double support duration), suggesting a positive effect on dynamic balance, which may prevent falls in patients with PD.

Remarkably, while although differences in improvements of biomechanical parameters were found, no significant differences emerged with respect to UPDRS-III scores. This may suggest that clinical scales alone are not exhaustively sensitive in determining changes in some motor aspects induced by physiotherapy and, thus, must be supplemented by objective instrumental measures.

However, given that most patients were in stages 1 through 3 and only four patients were in stage 4, it is conceivable that our results from the PMR plus VC only support it as an effective method in patients in stages 1–3 H–Y. As such, we point out that our results may not be applicable to more severe cases of PD.

The main limitation of this study is the small sample size at crossover. Although the number of eligible individuals was relatively high, many patients were excluded due to transportation problems from the crossover portion of the study. The limitation of the small sample size at crossover, even given that it was partly offset by the adoption of sensitive quantitative measures of motion, suggests that the results should be interpreted with substantial caution. However, the crossover design, which evaluates intra-individual changes, still allowed the detection of a therapy response, which may have been missed in a similar sized parallel group study. Although the number of subjects at crossover did not meet the sample size criteria and thus did not allow for the same inferential statistics used in the main portion of the study, we still found some significant improvements with treatment A compared to treatment B (Figure 3). The trunk and right ankle RoM improved more with treatment A than with treatment B at T1 and T2. An important result from the crossover design was that no carryover effect was found after the washout period, suggesting that the effect of both treatments lasted no longer than 4 months.

Another possible limitation of this study is that it is difficult to conclude that either PMR alone or VC alone is better than conventional therapy. This study proposed using sensory cueing, which is a well-established technique for gait rehabilitation, as adjunctive treatment to the PMR system, within a unique rehabilitation program. We suggest that PMR treatment may result in globally improved trunk control, hip motion, strength, and endurance (in addition to other factors), predisposing patients to the improvement of the gait rhythm and automaticity induced by the use of the external VC.

However, VC was also an integral part of the conventional physiotherapy used in this study. The main difference was that in treatment A, the VC was systematically executed at the end of the PMR for 20 min, whereas in the conventional treatment, it was executed for 30 min and discretionally applied during the course of each treatment session. Although both treatment groups underwent VC, we cannot entirely explain or confirm the specific contribution of both the VC and PMR. For instance, the specific contribution of the VC could be different based on which rehabilitation treatment it was associated with. A three-branch trial design (conventional physiotherapy, PMR, and VC treatments) is needed to understand the specific contribution of the PMR alone compared to either conventional physiotherapy or VC.

Despite these limitations, this study proposes a comprehensive rehabilitation treatment regime in addressing key pathological outcomes of PD. Furthermore, the results are consistent and can be generalized to clinical practice. However, further studies are needed to assess the long-term effect of this rehabilitative approach.

In conclusion, the present findings show that PMR plus VC is effective in improving gait performance, balance, and trunk control and should be considered as a possible rehabilitative strategy for the treatment of PD and other neurodegenerative diseases.

## Data Availability

All datasets generated/analyzed for this study are included in the manuscript and/or the Supplementary Files.

## Ethics Statement

This study was carried out in accordance with the recommendations of name of guidelines, name of committee with written informed consent from all subjects. All subjects gave written informed consent in accordance with the Declaration of Helsinki. The protocol was approved by the name of committee.

## Author Contributions

MS contributed to the study design, revision, and manuscript elaboration. MP, DG, GS, and SC were in charge of the patient's enrollment and rehabilitation. GM, SC, FP, ES, and MB were in charge of the supervision and manuscript elaboration. GC, AR, and CC were in charge of data analysis, statistical analysis, and manuscript elaboration.

### Conflict of Interest Statement

GM, ES, and SC are involved with the “Riequilibrio Modulare Progressivo (RMP)” association and received fees for teaching in post-graduate classes. The remaining authors declare that the research was conducted in the absence of any commercial or financial relationships that could be construed as a potential conflict of interest.

## Supplementary Material

Figure S1 This figure illustrates the mean and the standard deviation values of all 40 patients considered for treatment at the three evaluations (T0, T1, T2). Asterisks (^*^) denote statistically significant differences.
