# Supplementary figures and images for: Progressive Modular Rebalancing System and Visual Cueing for Gait Rehabilitation in Parkinson's Disease: A Pilot, Randomized, Controlled Trial With Crossover
Source: Front Neurol. 2019 Aug 29;10:902. doi: 10.3389/fneur.2019.00902 (PMC6730596; doi:10.3389/fneur.2019.00902)

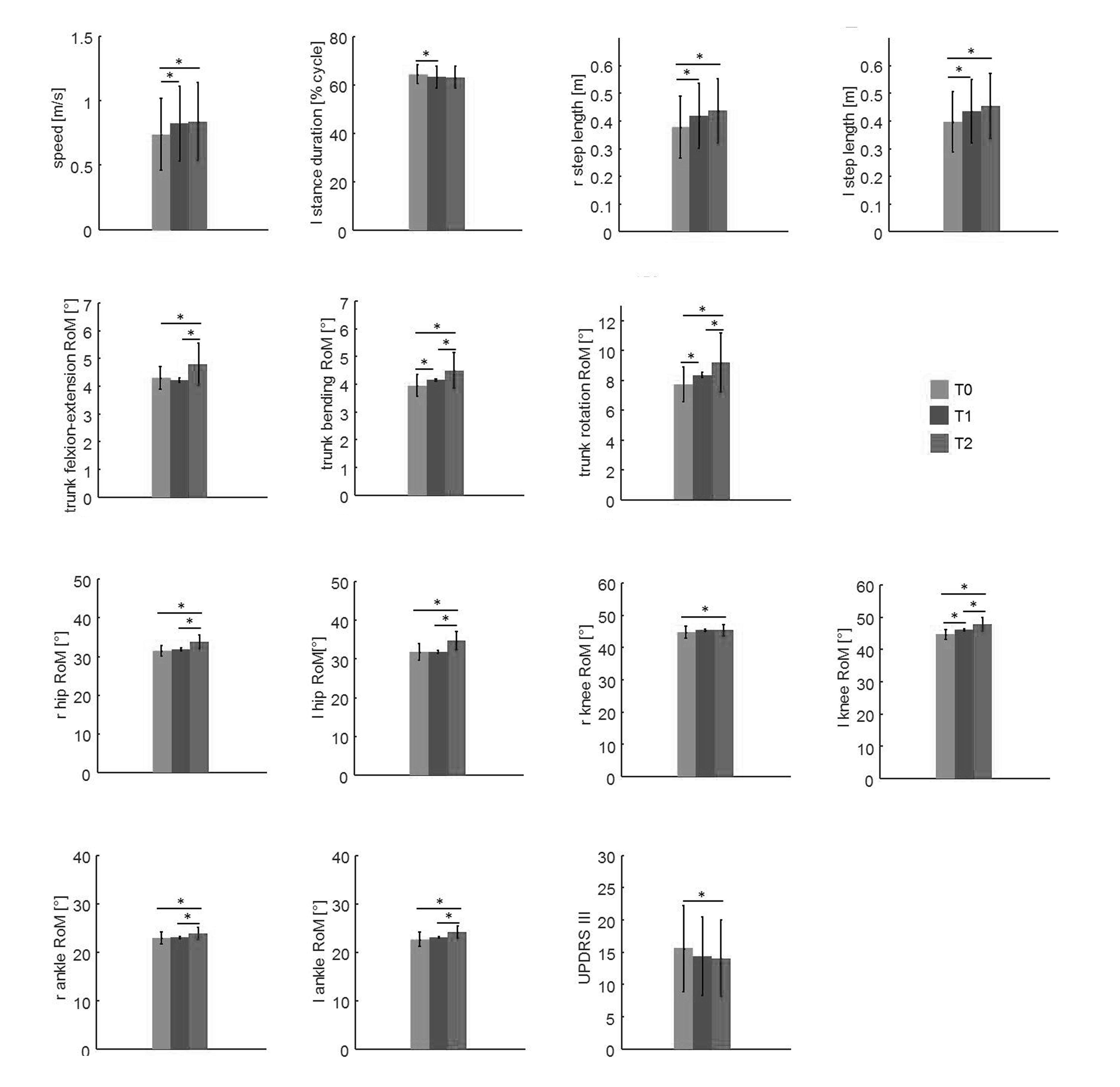

Supplement: Figure S1 — This figure illustrates the mean and the standard deviation values of all 40 patients considered for treatment at the three evaluations (T0, T1, T2). Asterisks (*) denote statistically significant differences. [file Image_1.TIF]
